# Esthetic Dentistry on Twitter: Benefits and Dangers

**DOI:** 10.1155/2021/5077886

**Published:** 2021-12-08

**Authors:** Nesreen A. Salim, Fahed Jubair, Yazan M. Hassona, Sarah Izriqi, Dana Al-Fuqaha'a

**Affiliations:** ^1^Prosthodontic Department, School of Dentistry, The University of Jordan. Consultant in Fixed and Removable Prosthodontics, The University of Jordan Hospital, Amman, Jordan; ^2^Computer Engineering Department, School of Engineering, The University of Jordan, Amman, Jordan; ^3^Oral Medicine and Special Care Dentistry, The University of Jordan, Amman, Jordan; ^4^School of Dentistry, The University of Jordan, Amman, Jordan

## Abstract

**Objectives:**

The demand for esthetic dentistry is growing, and social media constitute the main driving force behind this revolution. Twitter is a leading social media platform; however, there is a lack of research on the pattern of communications and the impact of Twitter on esthetic dentistry. The purpose of this study was to analyze the content of tweets related to esthetic dentistry and to investigate how Twitter has affected the perception of, and demand for, esthetic dentistry. Moreover, it aimed to assess the potential benefits and risks of esthetic dentistry advertising strategies on Twitter as a potential social media platform.

**Materials and Methods:**

Using a specialized web scrapping tool made available by the Python programming language, a total of 54,000 public tweets were downloaded from Twitter at real-time by matching specific keywords. The downloaded tweets were manually filtered, and 13,787 tweets were included and categorized into four tweet sets by content.

**Results:**

The main categories were tweets regarding specific cosmetic procedure, totaling 56.53% (43.2% for veneers, 13.3% for whitening), advertisements (36.72%), information (5.53%), and general cosmetic dentistry (1.22%). Negative opinions towards veneers and whitening were the most common themes (68.03% and 43.44%). For veneers, illegal use for kids was the most common negative theme (59.7%) and being destructive for whitening (86.3%). Positive opinions counted 6.26% of veneers and 4.3% of whitening tweets. Advertising for whitening products was the second most common between all tweets (16.6%), and advertising for dental practices was the third (14.7%). Presenting facilities/services was the most common marketing strategy for dental practices (49.7%). Twitter was weakly used to circulate educational content (5.5%) and to post reviews (0.75%).

**Conclusion:**

Twitter was extensively used to share experiences/opinions towards dental cosmetic interventions. Advertising is another vital use of this platform. However, circulating information was the least common use of this social media. Additionally, tweeters were rarely to post online reviews and specific advices and recommendations regarding esthetic dentistry. Moreover, females tended to participate and tweet more significantly in cosmetic-related topics than males. This platform should be tailored efficiently to target users' priorities and to improve health literacy and self-care. Twitter was weakly used to circulate educational content according to this study and rarely used to share online reviews.

## 1. Introduction

Social media have a huge impact on the way people communicate ideas and information and have revolutionized an interactive two-way sharing of ideas using online networking [1–3].

The interactive nature of social media enables patients and caregivers to obtain health-related information while also contributing to a networked process of content development and circulation [4]. Previous studies showed that patients and their caregivers use social media as a way to share their illness experiences [3–5] and engage in health-related discussion with both the general public and within scientific communities [6,7].

Several studies showed that social media platforms including Facebook, YouTube, Twitter, and Blogs have a significant impact on perception, practice, and attitudes of both patients and healthcare professionals in various medical and dental fields [8,9]. Social media platforms have been used in education, advertisement, self-promotion, and marketing [3]. The increasing use of social media has a great impact on communication patterns of healthcare-related issues worldwide [3,10–12].

Twitter is a well-known online social networking platform that currently has 330 million monthly active members and enables users to send short 280-character messages called tweets [13]. Certainly, there has been wide recognition that Twitter is a powerful gauge of public sentiment across a range of current social and healthcare issues such as opioid abuse, public perception of immunizations, perceptions of e-cigarettes, trending infectious disease, cancer, and dental pain [6,14–17].

The impact of social media in dentistry has been demonstrated in various “dental” fields including oral cancer, dental trauma, dental implants, root canal treatment, gingival recession, networking, advertising, and recruitment [11,17–19]. In the present study, we wanted to investigate the impact of a social media platform, specifically Twitter, on the demand for esthetic dentistry. Specifically, we aimed to characterize and analyze the content of tweets related to esthetic dentistry and to investigate the manner in which Twitter has affected the perception of, and the demand for esthetic dentistry, and the potential benefits and dangers of advertising strategies for esthetic dentistry on Twitter as a potential social media.

To the best of our knowledge, this is the first study to investigate Twitter content related to esthetic dentistry.

## 2. Materials and Methods

### 2.1. Study Design

This cross-sectional study explored the cosmetic dentistry-related communications by investigating different aspects of messages posted via the microblogging services “Twitter.” Day-to-day patterns of communication pertaining to cosmetic dentistry were characterized and investigated.

### 2.2. Data Collection

**Figure 1:**
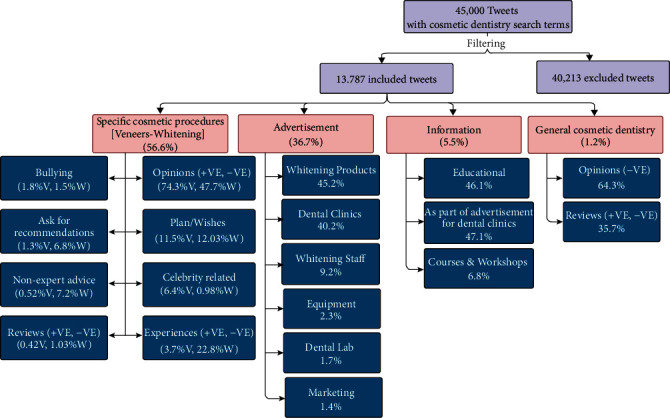
Flowchart showing the percentages of the primary categories (pink-shaded) and the primary subcategories (dark blue-shaded).

**Figure 2:**
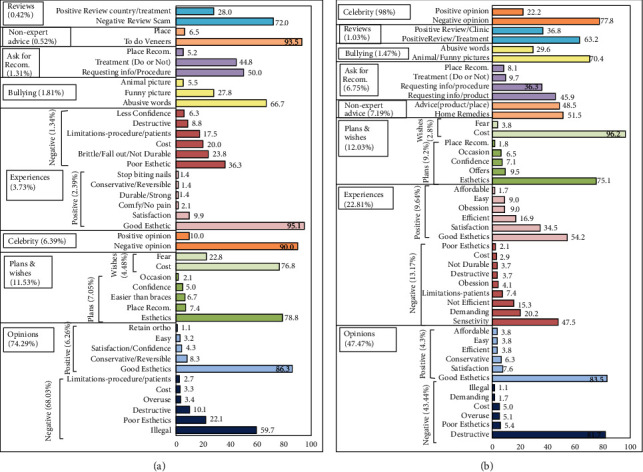
Categorical bar charts presenting percentages of primary (vertical-oriented titles) and secondary subcategories (horizontal-oriented titles) in (a) veneers and (b) teeth-whitening (Recom: recommendations and Info: information; all numbers are presented as percentages).

**Figure 3:**
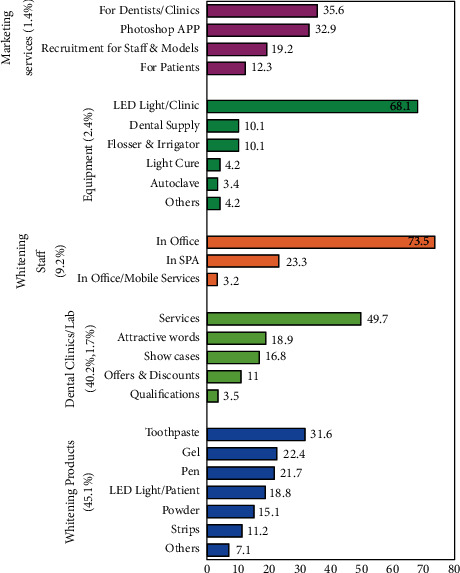
Categorical bar chart presenting percentages of primary (vertical-oriented titles) and secondary subcategories (horizontal-oriented titles) in advertisement main category.

A large sample of English tweets were collected from 24^th^ February to 12^th^ March 2020 using a web scrapper tool (the Twitter Python library (Tweepy version 3.10.0). The web scrapper tool used a *stream listener* instance to continuously track and download all public, real-time tweets with the keywords “veneers, lumineers, teeth bleaching, teeth whitening, cosmetic dentistry, esthetic dentistry and composite facing.” Then, the collected tweets underwent further analysis and were categorized by one investigator, resulting in dividing the tweets into four tweet sets (primary categories): (1) specific cosmetic procedures (veneers and whitening); (2) advertising; (3) information; (4) general cosmetic dentistry (Figure 1, Table 1). The content of each tweet within each main category was analyzed and coded manually and further subdivided according to its theme into primary and secondary subcategories according to their content (Figures 1–3). The gender, the number of likes, and the retweets for each tweet were extracted.

Tweets that were nonrelevant or written in non-English language were excluded from the study.

### 2.3. Data Analysis

All tweets were inserted into Excel spreadsheet, and the Python programming language was used to extract the frequencies for each tweet set in primary and secondary categories using substring search for relevant keywords in each category.

## 3. Results

A total of 54,000 tweets were collected, but 40,213 tweets were excluded because 33,110 tweets were irrelevant, 5,653 tweets were non-English, and 1,450 tweets were not available, resulting in a dataset of 13,787 dental cosmetic-specific tweets posted by 10,192 unique accounts.

The terms “veneers” and “whitening” were the most frequent matches in the designated search terms, accounting over 90.0% of the collected updates of the included tweets, and the term “lumineers” was the least frequent among other used terms (1.37%).

With regard to Twitter user type, 68.7% of the included tweets were posted by individuals (type 1), 17.4% by dental clinics/dentists (type 2), and 13.9% by advertising companies and magazines (type 3). The analysis of the individual users' characteristics revealed that 64.7% were females and 35.3% were males (Table 1).

In regard to the main categories, the majority of tweets were related to a specific cosmetic procedures category, totaling 56.53% (43.2% for veneers and 13.3% for whitening), followed by advertisements (36.72%), information (5.53%), and general cosmetic dentistry category (1.22%). The main categories and the primary subcategories are shown in Figure 1 and Table 1.

### 3.1. Specific Cosmetic Procedures Category

Both dental veneers and dental whitening were classified into 8 primary subcategories: opinions (+ve, -ve), experiences (+ve, -ve), asking for recommendations, nonexpert advice ± home remedies, bullying, celebrity related, plan/wishes, and reviews (Figures 1 and 2).

### 3.2. Veneers

Figure 2 presents the Twitter users' both positive and negative opinions towards dental veneers' use and practice and the secondary subcategories for each primary subcategory. Negative opinions were the most frequently posted tweets in the veneers category (68.03%) and the most frequent among all subcategories within other main categories (29.39%) (Tables 1 and 2). Moreover, illegal use of veneers for kids was the most commonly tweeted negative aspect of veneers (59.7%), followed by poor esthetics (22.1%), and big size and fake appearance were the most criticized aspects in esthetics (50.1% and 22.7%, respectively). Being destructive was the third most common negative aspect mentioned by tweeters (10.1%). In contrast, positive opinions represent 6.26% of the veneers category and 2.7% among all other subcategories, with esthetics improvement being the most positive thing tweeted relevant to dental veneers (86.3%).

On the other hand, tweets which share positive personal experiences (2.39%) were a little bit higher than tweets that expressed negative experiences (1.34%). Good esthetics was the most positive aspect experienced by tweeters (95.0%), followed by confidence and satisfaction (9.9%). On the other hand, poor esthetics was the most negative aspect experienced (36.3%), followed by high cost (20.0%) and being not durable and destructive (26.3%). 47 tweets were about clip-on veneers, and all expressed negative experience with ill fit and big size with poor esthetics.

A considerable number of tweeters plan already for or wish to have veneers (7.05% and 4.48%, respectively). The most common reason for planning to get veneers was purely for esthetics even if there is no actual need (78.8%). The main obstacle for tweeters who wish to have veneers was the high cost (76.8%), followed by fear of the procedure (23.2%).

Celebrity-related tweets represented the third most frequent subcategory in veneers category (6.39%). Negative opinions for celebrities' veneers represented 90.0% of this subcategory, and esthetics was the most frequent negative description (84.5%), followed by overuse (10.8%). Bullying tweets represented 1.81% of veneers tweets, using abusive words in 66.7%.

1.83% of veneers category tweets were asking for recommendation and nonexpert advice. Inquiring information about veneers as a cosmetic procedure was the most frequent request (50%), and to use veneers was the most given advice (39.5%).

Reviews were the least subcategory (0.42%), of which 72% were negative reviews. Scam fraud of a product “clip-on veneers” was solely the reported negative review (100%). The positive review was mainly related to a specific country (71.4%) with Colombia being the most frequently mentioned country, and 28.6% was obtaining satisfactory treatment.

### 3.3. Whitening

Similar to veneers' results, the tweeters' both positive and negative opinions represented the most frequent subcategory within the whitening category (47.74%), with negative opinion for dental whitening being the most common (43.44%), and the fourth most common among all other subcategories within the main categories (5.78%) (Table 1). Moreover, the most common negative aspect tweeted of dental whitening was being destructive to tooth structure (86.3%), and esthetics was the most positive thing relevant to dental whitening among other positive aspects (83.5%), followed by satisfaction and confidence (7.6%, Figure 2).

On the other hand, tweets which share both positive and negative experiences accounted for the second most frequent subcategory within the whitening category (22.81%). However, in contrast to the veneers category, negative personal experience tweets (13.17%) were much higher than the positive ones (9.64%). Sensitivity was the most negative aspect experienced by tweeters (47.5%), and whitening strips were reported in 66.1% of these tweets. Demanding treatment was the second negative aspect (20.2%), and long procedure was the main complaint in these tweets (51.0%). However, esthetics and confidence were the most positive aspects experienced (88.7%).

A significant number of tweeters plan already for or wish to do dental whitening (9.20% and 2.83%, respectively). The most common reason for planning for whitening was esthetics even if there is no actual need (75.1%). The main obstacle for tweeters who wish to do whitening was the high cost (96.2%).

Celebrity-related tweets regarding whitening, in contrary to veneers, were the least frequent subcategory within the whitening category (0.98%). Negative opinions about celebrities' whitening were the most frequent description (77.8%) in this subcategory, and fake esthetics was the most common description in these negative tweets (78.6%), followed by overuse for this intervention (21.4%). The positive opinions for celebrities' whitening (good esthetics) represent only in this subcategory (22.2%). Bullying tweets represents 1.47% of whitening tweets, using mostly funny or animal pictures (70.4%).

13.94% of whitening category tweets were asking for recommendation and nonexpert advice (6.75% and 7.19%, respectively). Asking about whitening products was the most common request (46.0%), using different home remedies were the most given advice (51.5%), and celery was the most recommended home remedy (42.6%). Mushroom, sesame seed, broccoli, and green tea were also recommended as home remedies for teeth-whitening (41.2% for each) and many other products. Reviews for whitening were minimal (1.03%), and all posted reviews were positive (100%), which were mainly related to a satisfying treatment (63.2%).

### 3.4. Advertisement Category

The most commonly tweeted advertisements tended to be whitening products advertisements (45.14%), and it was the second most frequent subcategory between all other subcategories within the main categories (16.57%, Table 1). The second most frequent advertisement was advertising for dental clinics (40.16%), and it represents the third most common subcategory compared to all other subcategories within the main categories (14.75%). On the other hand, the least number of advertisements were for websites to support dentists, booking systems (Internet services), and Photoshop Applications (1.46%, Figure 3).

Within whitening products' category, whitening toothpastes were the most commonly advertised product (24.7%), followed by whitening gel and pen (17.5% and 16.9%, respectively). Additionally, the advertising pattern for clinics varied; advertising services and technologies were the most common (49.6%), followed by using attractive words (18.9%) and showing photos of treated cases (16.8%) (Figure 3). 

### 3.5. Information Category

Tweets with informational content were either educational or reports as part of advertisement for dental clinics (47.12%), purely educational/reports (46.06%), and for courses/workshops (6.82%, Table 3).

By combining both sources for educational content, the most common form of educational material was professional information reported by doctors (58.0%), followed by magazine articles (28.5%). The only theme for posted reports was illegal practice of whitening by beauticians (100%).

Whitening was the most common topic in this category (40.81%), followed by general cosmetic dentistry (32.81%) and dental veneers (26.38%, Table 4).

### 3.6. General Cosmetic Dentistry Category

Cosmetic dentistry in general was the least mentioned topic by tweeters (1.2%). Negative opinions towards cosmetic dentistry were the most commonly shared content (64.3%), followed by patient's reviews, of which 96.7% had positive reviews and good experience with cosmetic procedures. 91.7% of the negative opinions were criticizing the common illegal practice of cosmetic dentistry by nonlicensed personnel and 7.4% criticizing the overuse of clients for these procedures.

### 3.7. Results according to Gender, Type, Number of Likes, and Retweets

The most common themes posted by each gender were negative opinions for veneers (44.76% for females and 38.54% for males), followed by whitening products advertisements containing tweets by females (10.67%) and negative opinion for whitening procedure by male tweeters (15.05%). However, whitening advertisements containing tweets by males represent 6.6% of all males' tweets, and plan for veneers represents the third most common tweets by females (5.02%). Subcategories of the main information category were the least theme posted in tweets by females and males (0.73% and 2.09%, respectively), such as courses and information about cosmetic dentistry in general (Table 1). 

The most common themes posted by type 1 were negative opinions towards dental veneers (42.6%), advertisement of whitening products (9.3%), and negative opinions towards dental whitening (8.3%). In comparison, among the tweets relating to type 2, advertisement for dental clinic (70.1%) was the most common, followed by whitening products (6.1%) and information (5.4%). For type 3, advertising for whitening products was the most common (72.3%), followed by advertising for equipment (6.2%) and information about whitening (3.9%).

The themes of the most liked tweets were negative opinions towards both whitening and veneers (20.2% for each), and the most liked tweet was “Yellow teeth are stronger, the natural color of our teeth is a light yellow color. Whitening your teeth can permanently weaken them.”

Similarly, the themes of the most retweeted tweets were negative opinions towards both dental veneers and whitening procedures (82.5% and 6.7%, respectively), and the most retweeted tweet was “I still cannot believe this is what's under veneers.” Different examples of posted tweets are presented in Table 5, portraying various themes and opinions.

In this study, many countries have been recommended in the tweets to do cosmetic procedures. Colombia was the most frequently mentioned place (48.4%), and Turkey was the second most common recommended place (19.4%).

## 4. Discussion

Patients' desires in attaining attractive smile resulted in extensively growing demand of dental cosmetic interventions, and this mainly attributed to the influence of media on social norms and expectations [9]. To our knowledge, this is the first study to analyze and characterize the pattern of communication about cosmetic dentistry using Twitter platform and to investigate the manner in which Twitter has affected the perception of, and the demand for, esthetic dentistry. Although the overall number of male and female tweeters are nearly equal on this platform [19], this result is in line with previous studies [11,20], explained by the emotional nature of females as they like to keep close ties and to gain social information. But, males are more inspired by skills to gain information [21].

“Twitter has turned me off from ever getting veneers or a rhinoplasty” is one of the tweets posted during the study period. It shows that media exposure does not always portray esthetic dentistry in a positive light. A previous study highlighted the negative impact of media on public's perception on dental treatment [9]. In this study, negative opinions towards veneers were the most common theme, and illegal provision of veneers for kids and preying on young people were the main judgment against this intervention. It is important to shed light on this important issue, where preadolescents and young teens are not suitable candidates for veneers considering having large pulp champers and the high risk of pulp exposure. Moreover, placing veneers too early will compromise the esthetics of the veneer, where the position of the gums and teeth may change with future growth and gaps between the veneers and gum may become visible over time due to oral and facial development [22]. And, even when teeth are damaged, more conservative options such as composite bonding should be considered [23,24]. This problem can be solved by defining strict laws and regulations to govern dental practices through dental boards and child protective agencies [24,25].

Fake appearance, debonding, overuse, being destructive, and very high cost of dental veneers are other common negative points that potentially affected tweeter's perception towards veneers. The effect of veneers on cosmetic improvement is a complex process, is multifactorial, and is highly subjective, as there are no golden standards for esthetics [23,26,27]. This may explain the findings of this study regarding judging veneers as unesthetic intervention by a considerable number of Twitter users. On the other hand, many tweeters are planning or wishing to have veneers to improve esthetics even when there is no actual need, which again confirms that there is no golden index for esthetics [22–24], and people now is interested in cosmetic dentistry more than ever [20]. Interestingly, in a previous study, patient's decision to undertake specific cosmetic intervention was significantly influenced by tooth conservation, length of procedure, and costs [23], and all these themes were highlighted in the tweets as negative aspects of dental veneers.

Destruction of tooth structure was reported in this study as a negative aspect of veneers, and the most retweeted tweet was negatively judging this aspect, as well as frequent debonding. Significantly increased failure rates and increased partial or complete debonding of the veneers were associated with veneers bonded to a greatly reduced tooth structure [28,29]. Sadly, this aggressive trend of preparation is becoming a common practice over time, leaving no options for future orthodontic and restorative care [23]. Overuse and improper application of these interventions should be replaced with more conservative restorative options such as composite veneers or mastering minimal preparations for indirect porcelain veneers [23], especially in esthetically conscious patients [30].

Additionally, destruction by whitening products was the most posted negative opinion for whitening. It has been reported that whitening results in significant change and roughness in enamel leading to weak tooth structure and makes it more susceptible to extrinsic staining [31], and again, this explains why tweeters had reported negative experiences after whitening as it is a demanding procedure and it affects their lifestyle negatively. Sensitivity was the most negatively experienced consequence of whitening by tweeters. Sensitivity is a common side effect of whitening, and data from many previous studies showed that a considerable number of patients up to 65% ended up with increased tooth sensitivity and some patients terminated the treatment because of discomfort [31–33]. Importantly, the method of delivery seemed to have an effect on the level of the experienced sensitivity and the bad taste felt [31,34], where strips were the most commonly reported method highly associated with sensitivity in this study. Additionally, a previous study showed that whitening using trays led to lower release of peroxides into saliva compared to strips [35], and this may explain the increased feeling of bad taste with this product.

One of the important factors for dental practices and product-advertising companies to increase their income and to engage more clients/patients is to have social media presence [11,19,36]. Moreover, the pattern of marketing strategies is potentially important in attracting patients [11], and it was shown that Twitter is the preferred platform for dental marketing by dentists and described as a virtual community for them [19,20]. In this study, presenting dental services, facilities, and technologies was the most common marketing strategy used by dental practices. Interestingly, it has been reported that this marketing strategy was the most important factor to choose a dental practice by patients [11], and in the same study, participants strongly agree that dental practice should have a social media platform. Moreover, showing cases was a very common method of dental advertisement in Twitter in the current study, which may enhance attracting patients by watching before-after images, because they provide esthetics and quality results that patients are aiming to [37]. On the other hand, presenting qualifications and offers/discounts were the least used dental advertising strategy in this study. However, patients in a previous study considered qualification of the dentist is an important factor to choose dental practice [11], but dental qualifications and degrees are not fully understandable by patients and consequently not a potential strategy to be used [38]. Offers were reported previously as a less attractive strategy by females but a more important factor for males [11,17,36].

Looking for health information is the third most popular online activity measured in national surveys, and more than 59% of Internet users of adult population are health information seekers [39,40]. Additionally, more than half of dental practitioners and a similar ratio of patients in a previous study said that social media helped them to be more educated [19]. In this study, information containing tweets was fairly low (5.5%), which may reflect the reduced use of professionals for Twitter as a main platform to share their knowledge, research, and published articles because of lack of time and ethical concerns [20], as well as the reduced circulation of informational content between tweeters because of the difficulty of their content [11]. Some patients do not consider social media in general as a reliable source of information about esthetic dentistry, where photos and cases are intentionally edited [20]. According to recent social media industry figures, Twitter currently ranks as one of the leading social networks worldwide based on active users [6,13]. As of the fourth quarter of 2019, Twitter had 152 million daily active users worldwide [41]. The Pew Research Center, which tracks social media usage among the United States adult Internet users, reported that Twitter usage has significantly increased from 18% to 23% over the past year, with a significant increase of 5%–10% in users older than 65 y [42]. Accordingly, Twitter should present a cost-effective and wide-reaching modality for providing educational content for different types of interventions and healthcare issues and should be tailored to target the priorities of users [11,17,19].

Criticisms of the use of social media in healthcare have also arisen. The availability of misinformation is a jeopardy, as healthcare providers are unable to guide and control the content that is posted or discussed [43]. Harmful results can also be resulted from inappropriate substitution of online information or advice for in-person visits to a healthcare provider, and this has been cited as a limitation of the use of the Internet generally and the social media in particular [44,45]. Negative usage of social media has also been highlighted in the context of confidentiality and professionalism, use by children and youth due to a limited capacity for self-regulation and susceptibility to peer influence, and promotion of high-risk products and services [18,46,47]. In this study, advertising an unlimited number of whitening products was the second most common use of Twitter and these advertisements should be handled cautiously and carefully, where people should find out if their products are properly regulated or attend their dentist for regulated professional treatment. What is important to understand is that the effects of whitening teeth with unregulated products can damage their teeth in the long term and lead to permanent irreversible damage to the enamel [31].

Noteworthy is that all posted reports as a source of information were solely about illegal whitening by beauticians, and the third most common advertisement next to whitening products and clinics according to this study was advertising for whitening staff. And, this reflects the negative side of social media, where these unlicensed personnel are preying and attracting clients through open connections with limited, if any, regulations. According to the General Dental Council (GDC), the dentistry practice of teeth-whitening “can only safely and legally be offered by registered dental professionals” and stated that failure to comply with the requirement to be registered can result in a criminal record and an unlimited fine [25].

The BBC carried out a recent investigation looking into the prevalence of illegal teeth-whitening in the UK and found a marked increase by 26%, when 732 cases of illegal teeth-whitening procedures were reported to the GDC in 2019 compared to 582 cases of illegal teeth-whitening from the year before [48]. The real number could be much higher because dentists' regulatory body relies on reports from customers. The rise of unregulated teeth-whitening products and services online, and in particular provided by nondentists, is a serious issue. People need to realize that whitening teeth is a healthcare procedure that needs to be governed by a healthcare professional and there can be repercussions including burns, blisters, and tooth loss, as warned by the BBC report [48].

In this study, tweeters were rarely to give online reviews through this platform (0.75% of all tweets). Reviews were reported by patients as an important factor to choose or to decline the treatment in a specific dental practice [11]. According to Forbes, negative reviews prevented 94% of clients from visiting a practical business [49], and on the other hand, patients and dentists can benefit from a good reputation of a dental practice/dentist [19]. Moreover, recommendations and advices were also very limited (1.5% of tweets), and again, this factor was defined by patients to be important to choose a treatment/dental practice [11]. Noteworthy is that all negative reviews regarding veneers were against scam companies that keep charging clients and all customers complaining of their ill-fitted clip-on veneers with poor services and intentional ignorance, but the most important thing was that they removed the option of leaving reviews on their websites so clients cannot see them beforehand. This is another important aspect that should be controlled and regulated on these social platforms.

### 4.1. Limitations

Twitter is a vital social media platform for the public to communicate health concerns and experiences and could efficiently afford healthcare professionals new strategies to expand their services. However, Twitter-based research has limitations. Issues involve data access restrictions (accounts set to private) and user sampling and filtering, as well as legal and ethical concerns. Another limitation is that tweets that have been collected should match specific criteria (certain keywords) and this could reduce the overall retrieved tweets. In this study, to overcome these limitations and to improve the quality of our work, a huge number of tweets were collected and repeated and retweeted tweets were filtered out.

## 5. Conclusions

Twitter has a remarkable impact on dental practitioners and the public's perception towards esthetic dentistry. This impact has both negative and positive effects on patient's perception. This study showed that Twitter is an important platform mainly for individuals to express their opinions and to share their experiences about specific cosmetic procedures. And, it is a main platform for advertising whitening products and dental practices, with presenting dental services and facilities being the most common marketing strategy. However, communication regarding cosmetic dentistry in general and circulating information were the least common use of this social media. Additionally, tweeters rarely post online reviews and specific advices and recommendations regarding esthetic dentistry. Moreover, females tended to participate and tweet more significantly in cosmetic-related topics than males. Twitter is a leading platform that could be tailored efficiently to achieve our goals. It is a tool to communicate, advertise, learn, and succeed if used appropriately, wisely, and professionally.

## Figures and Tables

**Table 1:** Distribution and subcategories of tweets.

Main categories		General (%)	Gender		Type		
			Males (35.27 %)	Females (64.73 %)	Type 1 (68.73 %)	Type 2 (17.42 %)	Type 3 (13.85 %)
Specific cosmetic procedures	Veneers	43.21	57.96	65.14	62.62	0.04	1.26
	Whitening	13.32	25.32	15.54	18.98	0.25	1.68
Advertisement		36.72	12.60	16.99	15.44	78.77	89.41
Information		5.53	2.09	0.73	1.21	20.90	7.6
Cosmetic dentistry		1.22	2.03	1.6	1.75	0.04	0.05

The distribution of tweets according to the main categories in general, by gender and type, is provided. Type 1: individuals; type 2: dental clinics and whitening staff; type 3: companies and magazines.

**Table 2:** The most frequent ten subcategories in general and according to gender regardless of their main category.

General		Male		Female	
Code	Percentage	Code	Percentage	Code	Percentage
Negative opinion (veneers)	29.40	Negative opinion (veneers)	38.54	Negative opinion (veneers)	44.77
Advertising for product	16.57	Negative opinion (teeth-whitening)	15.05	Advertising for product	10.68
Advertising for clinic	14.75	Advertising for product	6.64	Plan to do (veneers)	5.02
Negative opinion (teeth-whitening)	5.79	Positive opinion (veneers)	4.40	Negative opinion (teeth-whitening)	4.66
Whitening staff	3.39	Celebrity-related (veneers)	4.22	Celebrity-related (veneers)	3.90
Plan to do (veneers)	3.05	Plan to do (veneers)	3.35	Positive opinion (veneers)	3.65
Celebrity-related (veneers)	2.76	Advertising for clinic	2.96	Whitening staff	3.06
Positive opinion (veneers)	2.71	Wish to do (veneers)	2.51	Wish to do (veneers)	2.98
Wish to do (veneers)	1.94	Whitening staff	1.97	Negative experience (teeth-whitening)	2.89
Negative experience (teeth-whitening)	1.76	Negative experience (teeth-whitening)	1.92	Advertising for clinic	2.51

**Table 3:** Distribution of tweets and information.

Information subcategories	General (%)	Gender		Type		
		Males (35.27 %)	Females (64.73 %)	Type 1 (68.73 %)	Type 2 (17.42 %)	Type 3 (13.85 %)
Only educational	46.06	64.28	66.68	65.21	20.89	86.89
Educational information as part of advertising	47.12	35.72	24.44	31.31	61.55	9.66
Courses and workshops	6.82	0	8.88	3.48	8.56	3.45
Total	**100**	**100**	**100**	**100**	**100**	**100**

The distribution of tweets according to the information category in general, by gender and type, is provided. Type 1: individual; type 2: dental clinics and whitening staff; type 3: companies and magazine.

**Table 4:** Distribution of information categories according to source and topic.

Source of information		Topic of information		
		Whitening (40.81 %)	Cosmetic (32.81 %)	Veneers (26.38 %)
Educational^*∗*^ (87.01%)	Dental blog/dental chat (11.81%)	4.82	24.88	10.00
	Professional (video, interview, article, lecture) (50.39%)	54.66	40.80	52.80
	Magazine article (24.81%)	22.84	22.88	28.80
Reports^*∗*^ (6.17%)	Illegal dental practice	15.11	0	0
Courses and workshops (6.82%)		2.57	8.40	11.44
Total		**100**	**100**	**100**

^*∗*^The information as only educational and as part of advertisement was combined.

**Table 5:** Examples of tweets extracted by matching designated search terms.

Tweet	Primary category	Secondary category
“She got the veneers a tad too long. Looks as if she borrowed them from a horse”/“you look like an alligator with veneers”	1. Specific cosmetic procedure category (veneers)	Bullying for veneers/funny picture-abusive words
“Megan's teeth have more veneers than a kitchen”	1. Specific cosmetic procedure category (veneers)	Celebrity/negative opinion veneers/overuse
“I cannot wait until I can afford veneers because my teeth insecurity gets worse every day”	1. Specific cosmetic procedure category (veneers)	Wish to have veneers/cost
“Looking at all these people with these extra-large veneers will make you extremely grateful for having naturally nice teeth”/” if elected president, I will make veneers illegal”/“anyone who has had their teeth taken (replaced with veneers) has also had their soul taken”	1. Specific cosmetic procedure category (veneers)	Negative opinion for veneers/overuse/size/esthetics
“I like them veneers over diamonds any day”	1. Specific cosmetic procedure category (veneers)	Positive opinion for veneers
“Getting veneers was the best decision of my life”	1. Specific cosmetic procedure category (veneers)	Positive experience for veneers
“My zoom whitening came out amazing my teeth are so white I am getting stopped on the street asking if they are veneers”	1. Specific cosmetic procedure category (whitening)	Positive experience for whitening/treatment
“You use any whitening products? Your teeth look great!”	1. Specific cosmetic procedure category (whitening)	Positive opinion for whitening/esthetics
“I wish I could snap my fingers and my teeth would automatically be white. Not a fan wearing these whitening strips”/“I put my whitening strips last night and my teeth hurt soooooo bad like I feel like my teeth are about to fall out”	1. Specific cosmetic procedure category (whitening)	Negative opinion/experience about teeth-whitening
“Did you know your teeth are often the first thing people see when you first meet them? Make a great first impression”	2. Advertising category	Advertise product/whitening pen
“A smile is worth a thousand words”/“beautiful smiles are created Here”/“Do you want porcelain veneers without drilling”	2. Advertising category	Advertise clinic/attractive words advertise clinic/services
“ if you try an over-the-counter whitener, make sure it has the american dental association seal of approval to ensure it has been tested and deemed safe for at-home use”	3. Information category	Educational/professional information
“Unbelievable professionalism! from the staff to the doctors. Made me feel at ease and relaxed. During the procedure I felt no pain at all, they were really concerned about my comfort.”	4. General cosmetic dentistry category	Positive review

## Data Availability

The data (original data) used in this study are available from the corresponding author upon request.
